# Strain-Dependent Inhibition of Erythrocyte Invasion by Monoclonal Antibodies Against *Plasmodium falciparum* CyRPA

**DOI:** 10.3389/fimmu.2021.716305

**Published:** 2021-08-10

**Authors:** Anne S. Knudsen, Kasper H. Björnsson, Maria R. Bassi, Melanie R. Walker, Andreas Kok, Bogdan Cristinoi, Anja R. Jensen, Lea Barfod

**Affiliations:** Centre for Medical Parasitology, Department of Immunology and Microbiology, Faculty of Health and Medical Sciences, University of Copenhagen, Copenhagen, Denmark

**Keywords:** *Plasmodium falciparum*, merozoite, PfCyRPA, inhibition, synergy, strain-dependence, blood-stage vaccine

## Abstract

The highly conserved *Plasmodium falciparum* cysteine-rich protective antigen (PfCyRPA) is a key target for next-generation vaccines against blood-stage malaria. PfCyRPA constitute the core of a ternary complex, including the reticulocyte binding-like homologous protein 5 (PfRh5) and the Rh5-interacting protein (PfRipr), and is fundamental for merozoite invasion of erythrocytes. In this study, we show that monoclonal antibodies (mAbs) specific to PfCyRPA neutralize the *in vitro* growth of Ghanaian field isolates as well as numerous laboratory-adapted parasite lines. We identified subsets of mAbs with neutralizing activity that bind to distinct sites on PfCyRPA and that in combination potentiate the neutralizing effect. As antibody responses against multiple merozoite invasion proteins are thought to improve the efficacy of blood-stage vaccines, we also demonstrated that combinations of PfCyRPA- and PfRh5 specific mAbs act synergistically to neutralize parasite growth. Yet, we identified prominent strain-dependent neutralization potencies, which our results suggest is independent of PfCyRPA expression level and polymorphism, demonstrating the importance of addressing functional converseness when evaluating blood-stage vaccine candidates. Finally, our results suggest that blood-stage vaccine efficacy can be improved by directing the antibody response towards defined protective epitopes on multiple parasite antigens.

## Introduction

Malaria is estimated to have caused 229 million cases and 409,000 deaths in 2019 according to the World Health Organization Malaria Report (1). The infectious disease is caused by *Plasmodium* parasites, of which *P. falciparum* is one of the most prevalent forms, accounting for the vast majority of malaria deaths. Since the millennium, there has been significant progress in reducing malaria mortality, however, the spread of antimalarial drug and insecticide resistance emphasizes the need for efficacious malaria vaccines to achieve control and elimination of disease (2).

The blood-stages of the *P. falciparum* life cycle, where merozoites invade and multiply within erythrocytes, cause the clinical manifestations of disease. Invasion of human erythrocytes is essential to parasite survival, and is the only time during blood-stage development when the parasite is extracellular and more vulnerable to direct antibody mediated inhibition. Blood-stage vaccines tend to target merozoite antigens and aim to prevent replication and development of clinical symptoms. To date, clinical trials of leading blood-stage antigens such as *P. falciparum* apical membrane antigen 1 (PfAMA1) (3, 4) and merozoite surface protein 1 (PfMSP1) have shown no significant efficacy despite inducing high antibody titers (5). The limited efficacy has been impeded by considerable sequence polymorphism in the target antigens (6), redundant invasion pathways (7) or insufficient magnitude and breadth for effective antibody mediated inhibition (8).

The binding of the promising merozoite vaccine candidate PfRh5 to the host receptor basigin is fundamental for parasite survival (9). PfRh5 forms a ternary complex with PfCyRPA and PfRipr. Although the exact function of the complex is unknown, it is associated with calcium influx into erythrocytes and it is essential for the following establishment of a tight junction between merozoites and erythrocytes (10, 11).

All proteins of the ternary complex exhibit low levels of polymorphism and are able to induce growth inhibitory antibodies [reviewed in (12)]. Here we focus on PfCyRPA, which constitutes the core of the ternary complex that stabilizes PfRh5 and PfRipr on either side (13). PfCyRPA is a 43 kDa protein with only one single-nucleotide polymorphism (SNP), R339S, above 5% prevalence (14). The protein is fundamental for erythrocyte invasion as conditional knockdown causes the loss of invasion activity (11, 15) and the protein has low sero-reactivity from natural exposure (16–18).

Crystal structures of PfCyRPA show that it adopts a 6-bladed β-propeller structure with five disulfide bonds, four intra-sheet and one inter-sheet (19, 20). More recently, a cryo-electron microscopy study of the ternary complex showed that blades 1, 4 and 5 of PfCyRPA provide contact sites for PfRh5 while blade 6 provides a contact site for PfRipr (13). The mechanism of action by which anti-PfCyRPA mAbs induce parasite growth inhibitory activities are to a large degree still unknown. A recent study has indicated that one anti-PfCyRPA mAb is capable of blocking PfRh5 from binding PfCyRPA, while other anti-PfCyRPA mAbs block neither PfRh5 nor PfRipr from binding, but still show similar growth inhibitory activities as the former (21). Thus, anti-PfCyRPA mAbs seem to induce growth inhibition of *P. falciparum* by different modes of action, which could be linked to their specific epitope or their kinetic properties.

Together with PfRh5, PfCyRPA has been identified as a promising blood-stage vaccine candidate (18, 21, 22). This is due to PfCyRPA being a highly conserved target that participates in a non-redundant invasion pathway (9, 14). Additionally, *P. falciparum* merozoite antigens, PfMSRP5, PfSERA9, PfRAMA, PfRipr and PfRh5 are able to induce growth inhibitory antibodies that in combination with anti-PfCyRPA antibodies have synergistic interactions *in vitro* (19, 22, 23). This suggests that combinations of merozoite antigens could be used in a rationally designed malaria vaccine to elicit synergistic polyclonal antibody responses.

In this study, we examine parasite neutralization utilizing a panel of PfCyRPA specific mAbs. Using a quantitative methodology, we explore the functional interplay between groups of mAbs and identify several synergistic combinations of mAbs, which will aid the development of blood-stage vaccine candidates. We also show that mAb combinations are effective in neutralizing short-term adapted parasite isolates from Ghanaian patients as well as heterologous laboratory-adapted *P. falciparum* strains, emphasizing the fundamental role of PfCyRPA in parasite growth. Additionally, the functional assessment identifies strain-dependent neutralization potencies, which our data suggest is independent of PfCyRPA expression level and polymorphism. These results emphasize the importance of addressing functional interactions of antibodies to pave the way for future blood-stage malaria vaccine designs.

## Materials and Methods

### Expression and Purification of Recombinant Proteins

The expression vector encoding the merozoite antigen PfCyRPA-bio-his (Addgene plasmid #50823) was a gift from Gavin Wright (Wellcome Sanger Institute, Cambridge, UK). The vector contained the 3D7 PfCyRPA DNA sequence encoding amino acids D29-E362 with three mutations introduced to remove N-linked glycosylations (S147A, T324A and T340A). Furthermore, the construct included a C-terminal region comprising ratCD4 (d3+4), followed by a biotinylation sequence and a hexahistidine (His6) tag (24).

Transient transfection of PfCyRPA-bio-his was performed using the Expi293F™ Expression System Kit (Gibco), as per manufacturer’s instructions. Supernatant was harvested five days post transfection for purification. The antigen was purified by IMAC chromatography using a 5 mL HiTrap IMAC HP column charged with 0.1 M NiSO_4_ x 6 H_2_O on the ÄKTAxpress system (both GE Healthcare). PfCyRPA was eluted in 500 mM imidazole in sodium phosphate buffer pH 7.2 and buffer-exchanged into PBS using an Amicon ultra centrifugal concentrator (Millipore). Quality of antigen was assessed using SDS-PAGE and western blotting using an anti-his-HRP (C-term) antibody (Miltenyi Biotec).

### Mice Immunizations

All animal studies were performed in accordance with the Federation of European Laboratory Animal Science Associations (FELASA) guidelines. The protocol was granted ethical approval by the Danish Animal Experiment Inspectorate (approval number: 2018-15-0201-01541). 6 week old, female BALB/c ByJR mice were purchased from Janvier Labs and used for immunizations. Five mice were immunized intramuscularly (i.m.) with 20 µg of PfCyRPA formulated in 50% v/v AddaVax adjuvant (InvivoGen), followed by two similar i.m. boosts at 2-week intervals. This was followed by a final intraperitoneal (i.p.) boost in PBS, 2 weeks later. Three days after i.p. injection, the mice were euthanized and spleens and blood samples were harvested.

### Production of Monoclonal Antibodies

Hybridomas were produced by fusing splenocytes of immunized mice with myeloma cells (SP2/0-Ag14) following the instructions and materials from the ClonaCell-HY hybridoma cloning kit (Stemcell Technologies). Washed splenocytes and myeloma cells were mixed and resuspended in polyethylene glycol (PEG), followed by aspirating the PEG solution and washing in ClonaCell-HY Medium B, before incubating the cells in ClonaCell-HY Medium C overnight (37°C, 5% CO_2_). The following day, cells were resuspended in selection medium, 10 mL ClonaCell-HY Medium C and 90 mL ClonaCell-HY Medium D, and plated out into 100-mm petri plates. Hybridoma cell lines were harvested 14 days post fusion and plated into 96-well culture plates in HT supplemented media.

### Enzyme-Linked Immunosorbent Assay (ELISA)

Antibody-producing hybridoma cells specific for PfCyRPA were identified by ELISA. Briefly, MaxiSorp flat-bottom 96-well ELISA plates were coated with recombinant PfCyRPA (2 µg/mL in PBS) overnight at 4°C. The plates were washed three times with PBS + 0.05% Tween20 (PBS-T), and blocked 1 h with 1% w/v casein in PBS. The blocking solution was removed and 50 µL hybridoma supernatant was added and incubated for 1 h. The plates were washed three times with PBS-T before incubation with an anti-mouse IgG (γ-chain specific) coupled to alkaline phosphatase (Sigma-Aldrich) for 1 h. After three washes with PBS-T, the positive wells were detected with 4-Nitrophenyl phosphate disodium salt hexahydrate tablets (Sigma-Aldrich) dissolved in 1x Diethanolamine Substrate Buffer (Sigma-Aldrich). Color development and absorbance were measured at 405 nm. PfCyRPA specific hybridomas were further single cell sorted using FACSMelody (BD) to obtain monoclonal cell lines.

### Antibody Production and Purification

For large-scale mAb production, hybridoma cell lines were cultured in 1000 mL Cell-Line bioreactor flasks (Sigma-Aldrich) as per manufacturer’s instructions. Monoclonal antibodies were purified by affinity chromatography using a 5 mL protein G sepharose column on the ÄKTAxpress system (both GE Healthcare). Antibodies were eluted in 0.1 M glycine pH 2.8 and immediately neutralized with Trizma hydrochloride solution 1 M pH 9. This was followed by buffer-exchange into PBS or RPMI 1640 using Amicon ultra centrifugal concentrators.

### Growth Inhibition Activity (GIA) Assay

The *P. falciparum* laboratory-adapted strains 3D7, FCR3, 7G8, Dd2 and K1 were maintained in culture using fresh O+ erythrocytes at 2% hematocrit. The culture medium was supplemented with 0.5% AlbuMAX (Life Technologies) as a substitute for human serum. *P. falciparum* clinical isolates from Ghana, P57 and P90, were maintained in similar culture medium with the addition of 2% v/v heat-inactivated human serum.

The ability of anti-PfCyRPA mAbs to inhibit *in vitro* growth of *P. falciparum* was assessed using the standardized GIA assay (25). Cultures were synchronized by incubation in 5% sorbitol one day prior to assay setup. On day 1 of the assay, synchronized mid-trophozoites were adjusted to 0.5% parasitemia and incubated for approximately 48 hours (depending on the *P. falciparum* strain) with various mAb and mAb combinations. Each mAb or mAb combination was set up in triplicate in half-area flat bottom 96 well plates (Corning). The one-cycle GIA assay was harvested when parasites reached early schizont stage. A biochemical measurement using the *P. falciparum* lactate dehydrogenase assay was used to quantify parasitemia, as previously described (25). The results of the GIA assays, expressed as percent growth inhibition, was calculated as follows:

$$GIA\;\%=100-[\frac{\left(A_{630}Inhibited\;sample-A_{630}RBCs\;only\right)}{\left(A_{630}Uninhibited\;sample-A_{630}RBCs\;only\right)}\times 100]$$

The percent of inhibition obtained from the test mAbs was compared with positive controls (anti-PfRh5 mAb R5.016 (26) and 5 mM EDTA) and negative controls (normal growth medium and a non-malaria specific mouse mAb).

### Assessment of Antibody Synergy

Analysis of drug combinations (in this case antibody combinations) have commonly been divided into effect-based and dose-effect-based approaches. The Bliss Independence model, here referred to as Bliss additivity, is an effect-based strategy that compares the effect resulting from the combination of two antibodies to the effect of the individual antibody at that specific concentration (27, 28). Bliss additivity defines synergy between two antibodies as an effect greater than what would be predicted from the two antibodies working independently. Contrary, antagonism is observed when the effect is less than the predicted additivity of the two agents.

In relation to the GIA assay where effect was expressed as percentage of growth inhibition. The predicted Bliss additivity can be calculated using the following equation (29):

$$GIA_{[A+B]Bliss}=\left(\frac{GIA_{A}}{100}+\frac{GIA_{B}}{100}-\frac{GIA_{A}}{100}*\frac{GIA_{B}}{100}\right)*100$$

The dose-effect-based approach considers that the proper way to compare combinations is to find what concentration of each antibody produces the same quantitative effect (e.g. EC_50_). By using Loewe additivity, the predicted additive effect of a combination depends on the individual dose-effect curves, which enable definitions of synergy, additivity and antagonism.

Loewe’s definition of additivity:

$$1=\frac{\left[A\right]}{EC_{50,A}}+\frac{\left[B\right]}{EC_{50,B}}$$

This equation also allows the calculation of the Combination Index (CI), $CI=\frac{\left[A\right]}{EC_{50,A}}+\frac{\left[B\right]}{EC_{50,B}}$. A CI < 1 indicates that the doses of antibody A and B, which produce a given effect in combination, are lower than the expected doses from additivity, which then can be interpreted as synergy. Likewise, a CI > 1 can be interpreted as antagonism, as the doses of antibody A and B, which produce a given effect in combination, are bigger than the predicted doses from additivity. This approach of assessing the outcome of drug combinations has been widely used in malaria research (22, 23, 29, 30), and furthermore has the advantage of enabling construction of an isobologram which allows an intuitive and graphical understanding of the effect of combining drugs.

### Surface Plasmon Resonance (SPR) Assays

Data was collected on a Biacore T200 (GE Healthcare). All data were reference subtracted from a reference cell (Fc 2-1, Fc 3-1 or Fc 4-1) and analyzed using the Biacore T200 Evaluation Software 3.0.

### Kinetic Analysis of PfCyRPA-Specific mAbs by SPR

Experiments were performed at 25°C in the HBS-EP+ running buffer (GE Healthcare). On a series S CM5 chip (Cytiva), an equal amount of rabbit anti-mouse IgG (Cytiva) was immobilized in all four flow cells using standard NHS/EDC chemistry. In the kinetic experiment, 100-150 RU of mAb was captured in Fc 2, Fc 3 and Fc 4, using a flow-rate of 30 µl/min for 30 sec. Next, six different concentrations of PfCyRPA (ranging from 30 nM – 0.12 nM) were injected in duplicate for 300 sec at 60 µl/min in all flow cells. The PfCyRPA analyte was >95% pure as assessed by SDS-PAGE and western blotting. A dissociation time was measured for 600 sec (3600 sec when necessary). Regeneration of the chip was performed using 10 mM glycine-HCl pH 1.7 for 25 sec. Specific binding of PfCyRPA to the captured mAb was obtained by subtracting both the reference flow cell (Fc 1) and a blank run with running buffer only. Sensorgrams were fitted to a global Langmuir 1:1 model, to determine the kinetic association and dissociation rate constants using the Biacore T200 Evaluation Software 3.0. Graphed data show the fold-change in affinity of each mAb to the reference protein 3D7 PfCyRPA relative to the affinity of each mAb to the PfCyRPA variant R339S. Fold-change lower than 1 were plotted as their inverse to allow more uniform data representation compared to fold-change higher than 1.

### Epitope-Binning by SPR

Experiments were performed at 25°C in the HBS-EP+ running buffer (GE Healthcare). Approximately 400 RU of active mAb was amine-coupled to a Series S CM5 chip (GE Healthcare) on Fc 2 (CyP1.9) and Fc 3 (CyP2.38) using standard NHS/EDC chemistry. The dual injection mode was used at a flow-rate of 20 µL/min to first inject 25 nM of recombinant PfCyRPA for 180 sec, directly followed by an injection of anti-PfCyRPA mAb at 30 nM for 180 sec over all flow cells. This was followed by a dissociation time of 180 sec and a regeneration step using 10 mM glycine-HCl pH 1.5 (GE Healthcare) for 5-20 sec, depending on the mAb immobilized on the chip. All data generated was subtracted by the reference flow cell (Fc 1) and blank runs with running buffer.

### Dot Blotting

Recombinant PfCyRPA (2 µg/mL) was prepared in PBS or PBS + 50 mM DTT. For analysis under reducing conditions, samples were heated to 95°C for 5 min prior to spotting onto a 0.45 µm nitrocellulose membrane and air dried for 10 min. The membrane was blocked in 5% skimmed milk in TBS + 0.05% Tween20 (TBS-T) for 1 h and washed twice in TBS-T. Afterward, the membrane was immersed into separate anti-PfCyRPA mAb solutions (5 µg/mL) in 2.5% skimmed milk in TBS-T for 1 h and washed three times. Bound anti-PfCyRPA mAbs were detected by incubating the membrane in an anti-mouse IgG (γ-chain specific) HRP-conjugated Ab (Sigma-Aldrich) solution diluted 3000-fold in 2.5% skimmed milk TBS-T for 1 h. After a final series of three washes, the dot blot was developed with the KPL LumiGLO Reserve Chemiluminescent Substrates (Sera Care) and images were obtained using the ImageQuant Las4000 (GE Healthcare).

### Generation of PfCyRPA Protein Fragments

The fragments of PfCyRPA were all based on the PfCyRPA-bio-his expression vector (Addgene, #50823). Primer pairs were designed to amplify different segments of the PfCyRPA DNA sequence, and included an overhang encoding the 5’ cloning site, NotI, or a 3’ cloning site, AscI. The amplicons were digested with the restriction endonucleases NotI and AscI (New England Biolabs) and ligated into the linearized PfCyRPA-bio-his expression vector. Like full-length PfCyRPA, the fragments comprised a C-terminal ratCD4 (d3+4), a biotinylation sequence and a hexahistidine (His6) tag.

The different expression vectors were transient transfected using Expi293F™ Expression System Kit (Gibco), as per manufacturer’s instructions. Supernatants containing the PfCyRPA fragments were harvested four days post transfection for purification. The fragments were purified by a IMAC chromatography using a 5 mL HiTrap IMAC HP column charged with 0.1 M NiSO_4_ x 6 H_2_O on the ÄKTAxpress system (both GE Healthcare). The fragments were eluted by a two-step approach (110 and 405 mM imidazole) in sodium phosphate buffer pH 8 and subsequently buffer-exchanged into PBS by Amicon ultra centrifugal concentrators (Millipore). Quality of antigens was assessed by SDS-PAGE and western blotting using an anti-his-HRP (C-term) antibody (Miltenyi Biotec).

Reactivity of anti-PfCyRPA mAbs to the recombinant fragments were assessed by ELISA, as described above with the following modifications. The purified mAbs were detected by anti-mouse IgG (γ-chain specific)-HRP (Sigma-Aldrich), and positive signals were detected by adding TMB substrate (ECO-TEK). The reaction was stopped with 0.2 M H_2_SO_4_, and the absorbance was measured at 450 nm.

### Protein Extraction From *P. falciparum* Merozoites

Late-stage parasites (40-44 h post invasion) were isolated by a magnetic-activated cell sorting (MACS) approach using a Vario-MACS magnetic separation unit and a MACS CS-column (both from Miltenyi Biotec). After isolation, the infected erythrocytes were resuspended in complete medium and incubated for additionally 5 h at 37°C. By filtering mature schizonts through 1.2 µm filters, the merozoites were mechanically released and isolated (31). The merozoites were washed in cold PBS, and the final pellet was resuspended in ice-cold RIPA buffer (Thermo Fisher Scientific) supplemented with a protease inhibitor cocktail (Roche Diagnostics). The merozoite pellet was fully disrupted by vigorous vortexing, followed by a 15 min incubation on ice. The lysates were cleared by centrifugation at 15,000 × g for 15 min and the supernatants stored at -80°C until use in SDS-PAGE.

### Quantitative Western Blotting on Merozoite Lysates

In order to normalize protein levels prior to gel loading, the protein concentration of merozoite lysates from three *P. falciparum* clones, 3D7, FCR3 and K1, was determined using the BCA protein assay kit (Pierce) as per manufacturer’s instructions. For SDS-PAGE, merozoites lysates and recombinant PfCyRPA, were resolved using NuPAGE 4-12% gradient Bis-Tris gels (Invitrogen) with MOPS running buffer. All samples were prepared in 6x sample buffer under non-reducing conditions and heated to 70°C for 10 min prior to loading. Proteins were transferred to nitrocellulose membranes in a wet blotting system as per manufacturer’s instructions (Novex X-Cell II, Invitrogen). After blocking the membrane, PfCyRPA was detected by mouse anti-PfCyRPA mAb CyP1.9, 5 µg/mL. The loading control *P. falciparum* heat shock protein 70 (PfHSP70) was detected by rabbit anti-PfHSP70 polyclonal Ab (SPC-186, StressMarq Biosciences) diluted 1:3000. Primary Ab’s were detected using HRP-conjugated rabbit anti-mouse immunoglobulin and goat anti-rabbit immunoglobulin respectively (both from DAKO). Western blots were developed using the LumiGLO peroxidase chemiluminescent substrate (SeraCare) and images were obtained using the ImageQuant Las4000 (GE Healthcare). The quantitative western blotting analysis was performed using the ImageQuant TL software, to calculate the relative abundance of PfCyRPA compared to PfHSP70.

### Intracellular Staining of PfCyRPA in *P. falciparum* Merozoites

The *P. falciparum* 3D7, FCR3 and K1 merozoites were prepared as described earlier (31). Magnetic purification using LS columns (Miltenyi Biotec) was performed to remove hemozoin from the isolated merozoites. The merozoites were resuspended in cold 3% BSA in PBS and seeded into 96-well U-bottom plates at roughly 4 × 10^5^ merozoites per 100 μl. To allow intracellular staining of PfCyRPA, merozoites were fixed in 4% paraformaldehyde in PBS for 15 min followed by permeabilization with 0.1% Triton X100 in PBS for 10 min. After overnight blocking in 3% BSA in PBS, merozoites were incubated with primary antibodies (25 µg/mL) at room temperature for 30 min followed by three wash steps with 3% BSA in PBS. Subsequently, merozoites were incubated with the secondary antibody (FITC conjugated horse anti-mouse IgG Antibody (H+L) 20 µg/mL (Vector Laboratories) for 30 min at room temperature. Nuclei were stained with DAPI (4’6-diamidino-2-phenylindole) at 300 nM for 10 min. Finally, merozoites were washed three times with 3% BSA in PBS and resuspended in 150 µl 3% BSA in PBS.

The samples were analyzed using the CytoFLEX S flow cytometer (Beckman Coulter) and data analysis was performed using FlowJo v10.7.1 software. Merozoites were gated by size and granularity in a SSC-A/FSC-A plot, followed by gating singlets in a SSC-H/SSC-A plot and finally by gating on DAPI fluorescence in a FL6-A(PB450)/FSC-A plot. Histograms were obtained using the FL1-A(FITC) channel intensity, followed by calculating the geometric mean.

### Statistical Analysis

To statistically assess the differences between the observed antibody combinations and the predicted Bliss additivity effect in the GIA assays, a two-way ANOVA with Bonferroni post-hoc testing was applied (29). A one-way ANOVA with a Tukey’s multiple comparison test was applied to compare the relative abundance or mean fluorescence intensity (MFI) of PfCyRPA between the three *P. falciparum* strains. All analyses were performed using GraphPad Prism version 9.0 and p-values < 0.05 were considered statistically significant.

## Results

### Strain-Dependent Variation in Anti-PfCyRPA mAb Neutralization

A panel of anti-PfCyRPA mAbs was generated by immunizing mice with recombinant full-length PfCyRPA. By PfCyRPA-specific serum IgG endpoint titer ELISA, the best immune responders were chosen, after which hybridoma cell lines were generated by fusing splenocytes of immunized mice with myeloma cells. Based on reactivity to recombinant PfCyRPA in ELISA, a panel of eight anti-PfCyRPA mAbs was generated.

**Figure 1:**
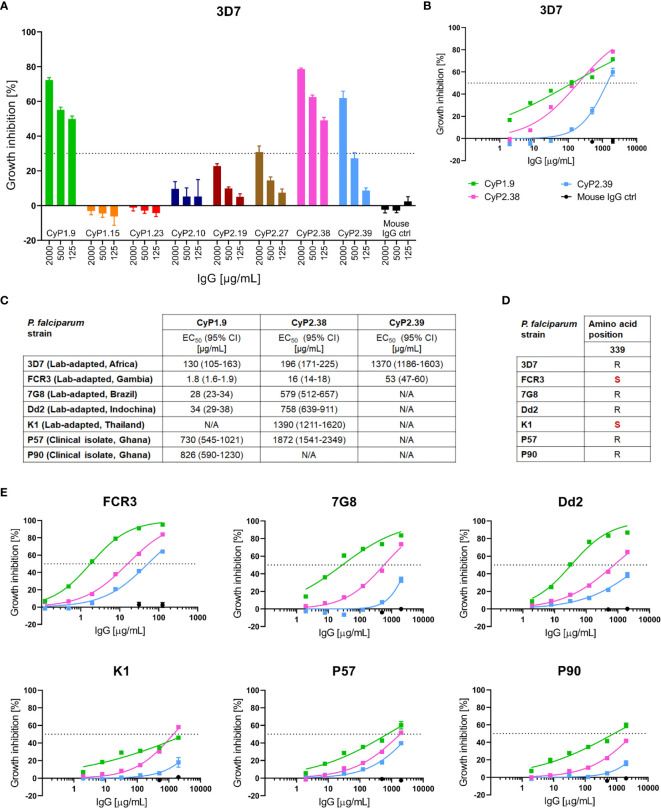
Growth inhibition activity of PfCyRPA-specific mAbs. **(A)** GIA assay on a panel of PfCyRPA specific mAbs tested at 2000, 500 and 125 µg/mL on *P. falciparum* 3D7 reference strain. The dotted line at 30% growth inhibition indicates the cut-off between mAbs inducing high levels of GIA and mAbs inducing negligible GIA. **(B)** GIA assay using dilution series of mAbs against 3D7 parasites. The data was fitted to a four-parameter dose-response curve to interpolate EC50 values. **(C)** Table of the interpolated EC_50_ values and 95% confidence interval (CI) of CyP1.9, CyP2.38 and CyP2.39 on all P. falciparum strains tested. EC_50_ values are referred to as not applicable (N/A) when exceeding 2000 µg/mL **(D)** Deviation from the 3D7 reference sequence (highlighted in red) at the most common polymorphic site of PfCyRPA, amino acid position 339 **(E)** GIA assays using dilutions series of mAbs on multiple laboratory-adapted and clinical isolate of *P. falciparum*. The data was fitted to a four-parameter dose-response curve to interpolate EC50 values. All GIA data points represent the mean of triplicates from three independent experiments. Error bars indicate SEM for all nine replicates over three experiments.

To characterize the ability of the anti-PfCyRPA mAbs to block merozoite invasion into erythrocytes, they were screened for *in vitro* growth inhibition activity (GIA) on the *P. falciparum* reference strain 3D7. The first screening was performed at three concentrations, 2000, 500 and 125 µg/mL, with a cut-off at 30% GIA, categorizing the mAbs into either “high-GIA” or “negligible-GIA” **(** **Figure 1A** **)**. Based on this grouping, dilution curves of only “high-GIA” mAbs were performed against the 3D7 strain to assess potency **(** **Figure 1B** **)**. By fitting the data to a four-parameter dose-response curve it was apparent that two of the mAbs (CyP1.9 and CyP2.38) had lower EC_50_ values than other anti-PfCyRPA mAbs previously described (18, 20, 32) **(** **Figure 1C** **)**.

To assess the strain-transcending capacity of the anti-PfCyRPA mAbs, GIA assays were performed on six heterologous laboratory-adapted and clinical isolates of *P. falciparum*. All strains originated from different geographical places and two of the strains, FCR3 and K1, contained the most common PfCyRPA polymorphism, R339S **(** **Figure 1D** **)**. The GIA assays showed strain-dependent variation in anti-PfCyRPA mAb potency, while maintaining a similar hierarchy of the three mAbs against all parasite isolates **(** **Figures 1C, E** **)**. FCR3 does in particular stand out, as it was more easily neutralized by all mAbs relative to 3D7, with EC_50_ values ranging from 1.8-53 µg/mL. In contrast, K1, P57 and P90 strains were less easily neutralized relative to 3D7, with EC_50_ values approaching the maximum assay concentration of 2000 µg/mL **(** **Figure 1C** **)**. Overall, the GIA assays indicate prominent differences in anti-PfCyRPA mAb potency despite the target being highly conserved.

### Anti-PfCyRPA mAbs Can Synergize When Used in Certain Combinations

**Figure 2:**
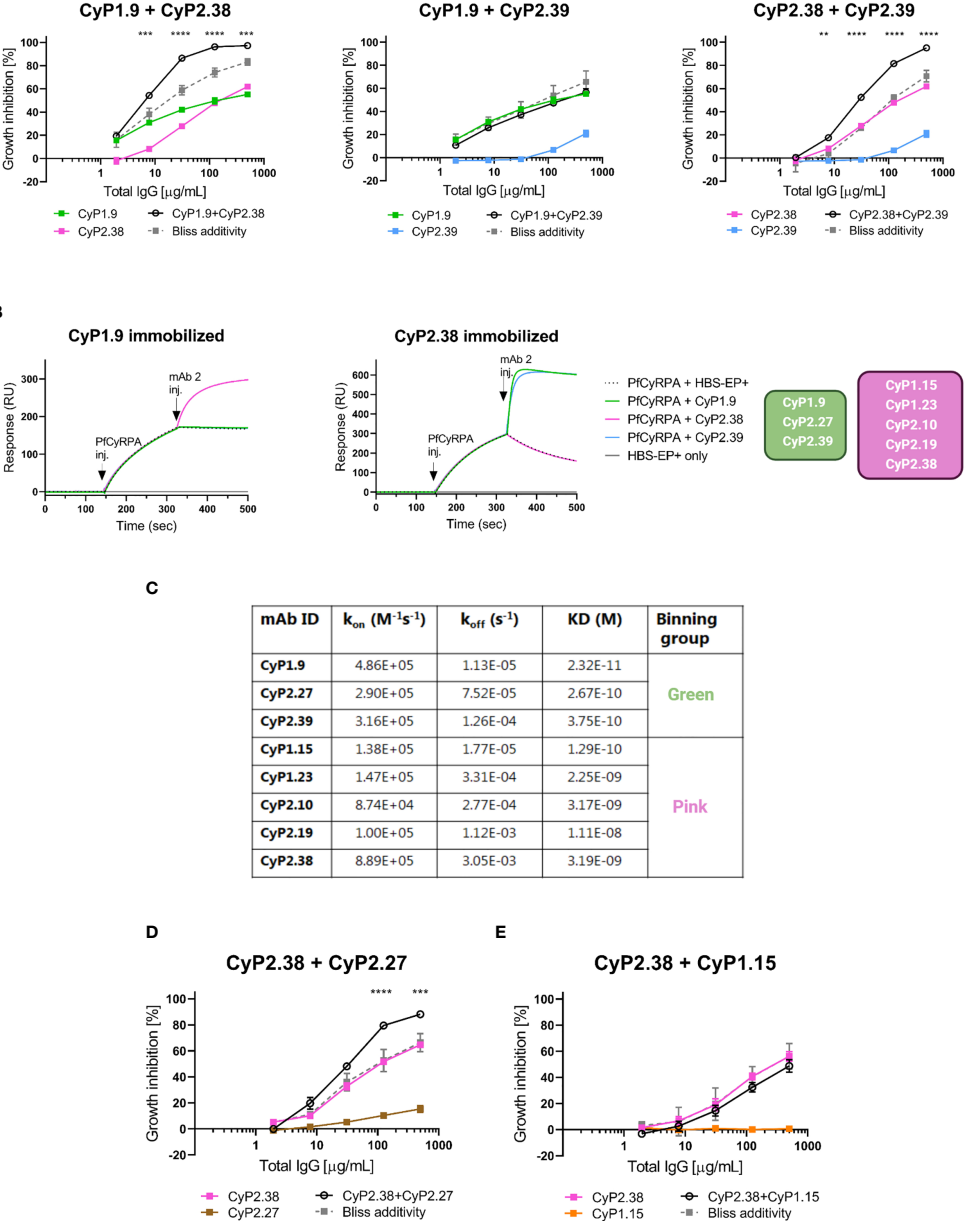
Association between growth inhibition activities of anti-PfCyRPA mAb combinations and binding site on PfCyRPA. **(A)** GIA assays using dilution series of individual mAbs and 1:1 combination of anti-PfCyRPA mAbs on 3D7 reference strain. Theoretical additive effects were calculated using Bliss additivity (described in Materials and Methods) and shown in the grey dotted line on each graph. A solid black line represents the experimental effect of mixing two anti-PfCyRPA mAbs. Data points represent the mean of triplicates from two independent experiments. Error bars show SEM for all six replicates over two experiments. The asterisks indicate where the experimental and predicted values of the mAb combination significantly show synergy, using a 2-way ANOVA with Bonferroni’s multiple comparison test (**p,0.01, ***p,0.001, ****p,0.0001). **(B)** Sensorgrams of epitope-binning experiments by classical sandwich blocking using SPR systems. The two most potent anti-PfCyRPA mAbs have been immobilized onto the surface of a CM5 chip, with one mAb in one flow cell. The first injection is the PfCyRPA antigen and the second injection is the competing mAb with both injections indicated by an arrow. An increment in RU at the second injection site is indicative of a non-competing mAb. **(C)** Kinetic properties of the panel of PfCyRPA mAbs in relation to epitope-binning group. Association-rate (k_on_), dissociation-rate (k_off_) and dissociation constant (KD) are listed. **(D)** GIA assay using CyP2.38 in combination with non-inhibitory mAbs from different or **(E)** same binning groups. Asterisks indicate where the experimental and predicted values of the mAb combinations are significantly different.

To determine if dual combinations of neutralizing mAbs could potentiate the neutralizing capacity, *in vitro* GIA assays were performed. For each combination of mAbs, the predicted additive GIA effect was calculated by the definition of Bliss Independence, here referred to as Bliss additivity (27, 29). For each combination, the experimental and predicted GIA were compared by a 2-way ANOVA test to statistically determine if the combination resulted in synergy, additivity or antagonism. Dilution curves were performed by combining two neutralizing mAbs in a 1:1 ratio against the 3D7 strain. It was evident that only certain combinations of anti-PfCyRPA mAbs were synergistic. CyP2.38 synergized with both CyP1.9 and CyP2.39, whereas the CyP1.9 and CyP2.39 combination was sub-additive (with the effect remaining as strong as the most potent individual mAb) **(** **Figure 2A** **)**.

To understand why only certain combinations of mAbs potentiate the neutralizing effect, epitope-binning experiments were conducted to further group them. The panel of anti-PfCyRPA mAbs was tested in a pairwise combinatorial manner by performing a classical sandwich blocking experiment using SPR **(** **Figures 2B** and **S1** **)**. By the classical sandwich approach, a mAb analyte was tested for binding to PfCyRPA that was captured *via* an immobilized mAb. This method defined two separate epitope bins shown in green and pink colors **(** **Figure 2B** **)**. In relation to the GIA assays on the anti-PfCyRPA mAb combinations, the results suggest that synergy is achieved only when different regions of PfCyRPA are targeted simultaneously.

Upon the epitope-binning experiments, it was observed that both bins contained mAbs with “high-GIA” and “negligible-GIA”. To better understand why only CyP1.9 and CyP2.39 (green bin) and CyP2.38 (pink bin) were neutralizing, kinetic parameters were assessed by SPR. The assay was setup by immobilizing polyclonal rabbit anti-mouse IgG on a CM5 chip, which captured anti-PfCyRPA mAbs followed by adding the recombinant PfCyRPA in a 3-fold dilutions series. Affinities were in the low nanomolar to high picomolar range for all mAbs **(** **Figure 2C** **)**, similar to previous findings (18). Of the mAbs in the green bin, CyP1.9 showed the fastest association-rate, slowest dissociation-rate, and hence the strongest affinity to PfCyRPA. Interestingly, this mAb also had the highest potency in the GIA assay. The other two mAbs in the green bin, CyP2.27 and CyP2.39, had very similar kinetics. However, CyP2.39 had a slightly faster association-rate than CyP2.27, which could explain the higher neutralizing capacity of CyP2.39. Of the mAbs in the pink bin, association-rate seemed to relate strongly with neutralizing capacity, as CyP2.38 recognized PfCyRPA the fastest and was the only inhibitory mAb in this bin.

After having grouped the mAbs by neutralizing capacity and epitope bin, we speculated whether GIA synergy could be obtained when pairing a “high-GIA” mAb with a “negligible-GIA” mAb from a different binning group. To test this, CyP2.38 (pink bin) was combined with CyP2.27 (green bin) and, as a control, also combined with CyP1.15 (pink bin). Dilution curves were setup by combining two mAbs at a 1:1 ratio against the 3D7 strain. Remarkably, synergy was achieved when combining mAbs from different binning groups, even when one component did not exhibit any GIA on its own **(** **Figure 2D** **)**. The results show the advantage of targeting multiple protective epitopes simultaneously, as the effect did not occur when combining competing mAbs **(** **Figure 2E** **)**.

### Identifying Protective Binding Domains on PfCyRPA

**Figure 3:**
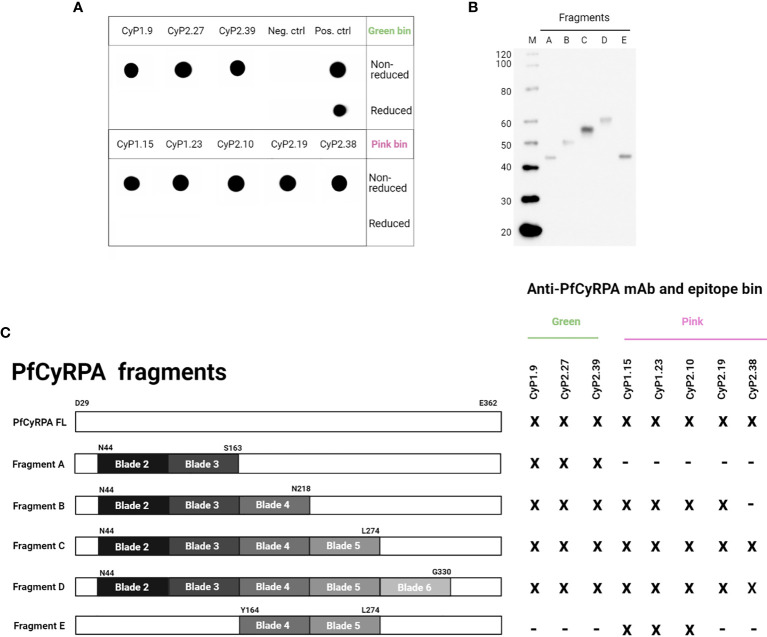
Assessing the protective bindingdomains of anti-PfCyRPA mAbs. **(A)** Dot blot showing anti-PfCyRPA mAbs binding to recombinant PfCyRPA in non-reduced or reduced conditions. The positive control is an anti-his (C-term)-HRP antibody, while the negative control is a secondary anti-mouse IgG-HRP antibody only. **(B)** Western blot of purified PfCyRPA fragments detected by an anti-his (C-term)-HRP antibody. **(C)** The reactivity of anti-PfCyRPA mAbs to purified fragments of PfCyRPA was assessed by ELISA and correlated to epitope-binning groups. x = reactivity, - = no reactivity.

The epitope binning assay simply determines which mAbs compete with each other; it does not provide any information about the epitopes. Thus, other methods were applied in order to characterize and identify the protective binding domains on PfCyRPA. To investigate if the epitopes recognized by the anti-PfCyRPA mAbs were linear or conformational, dot blots were performed using reduced or non-reduced recombinant PfCyRPA. Only non-reduced PfCyRPA was recognized indicating that all anti-PfCyRPA mAbs target a conformational epitope **(** **Figure 3A** **)**.

In addition, we tested the reactivity of anti-PfCyRPA mAbs to protein fragments of PfCyRPA by ELISA, with the aim of elucidating which binding domain they target. The different fragments were designed based on the three-dimensional 6-bladed β-propeller structure of PfCyRPA (19, 20), and were recombinantly produced in the Expi293F expression system as secreted proteins **(** **Figure 3B** **)**. Fragment A comprised blade 2 and 3, and served as a scaffold after which one blade was added at the time, except fragment E, which only comprised blade 4 and 5 **(** **Figure 3C** **)**.

All anti-PfCyRPA mAbs showed reactivity to fragment D **(** **Figure 3C** **)**, which lacked blade 1 of the full-length protein. Only the three mAbs from the green bin recognized fragment A, which includes the epitopes recognized by two already published anti-PfCyRPA mAbs (19, 20). The mAbs in the pink bin recognized fragments B and C, indicating that these mAbs target overlapping epitopes on PfCyRPA. The neutralizing and synergistic mAb, CyP2.38, showed reactivity to fragment C, suggesting that blade 5 of PfCyRPA is part of its protective epitope. Furthermore, the data also suggest that this conformational epitope is dependent upon the interaction between multiple blades on PfCyRPA, as CyP2.38 did not show any reactivity with fragment E **(** **Figures 3C** and **S2** **)**. The identification of binding domains shows that CyP1.9 and CyP2.39 target a region on PfCyRPA that has previously been shown to comprise protective epitopes, whereas CyP2.38 targets a novel protective epitope, which seems to depend on the proper tertiary structure of PfCyRPA.

### Assessment of Synergy by Loewe Additivity

Another way to assess the effect of combining mAbs is the isobologram analysis. This type of analysis provides a quantitative measurement of synergy by comparing the amount of drug required to achieve a certain effect when mixed, as compared to when used on its own. This approach uses the definition of Loewe additivity to measure the effect between drugs. Here, the definition was applied to the dual “high-GIA” anti-PfCyRPA mAb combinations against 3D7 parasites. The antibody interactions were assessed over a range of concentrations by a fixed-ratio method (30), where dose-response curves were constructed to interpolate EC_50_ values that were converted to the 50% fractional inhibitory concentrations (FIC_50_). For each different concentration ratio, the FIC_50_ values were used to construct an isobologram.

**Figure 4:**
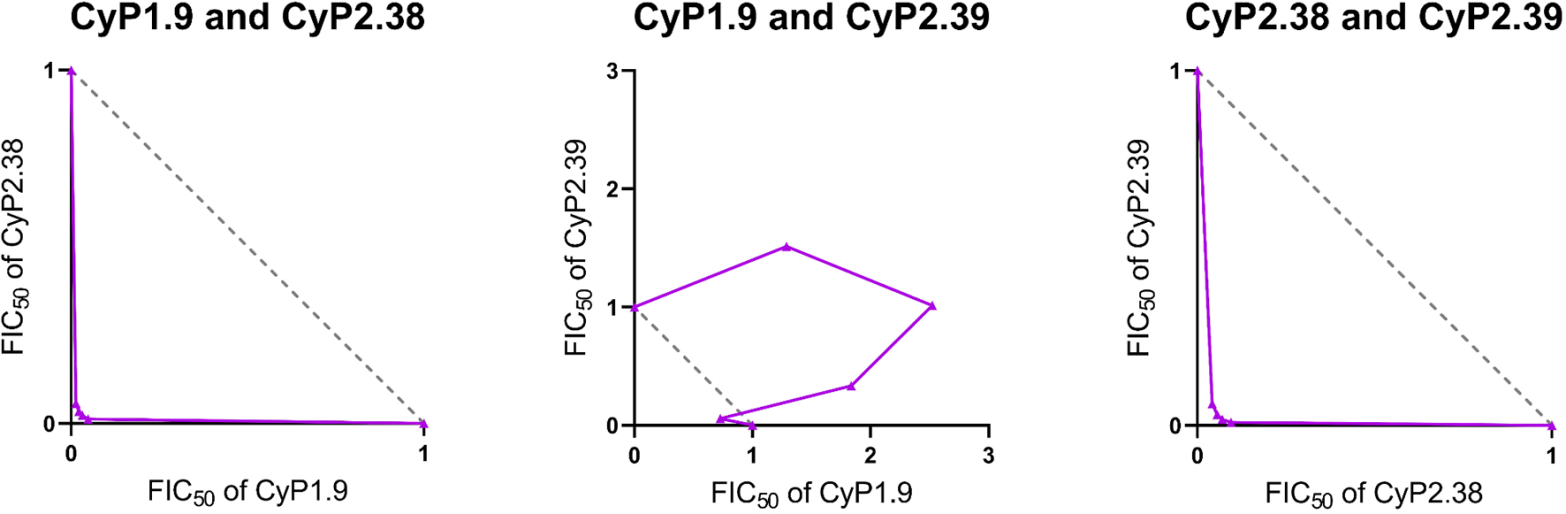
Isobologram analysis of GIA effects when combining anti-PfCyRPA mAbs. The isobolograms show the effect of combining anti-PfCyRPA mAbs using fixed-ratio mixtures (5:0, 4:1, 3:2, 2:3, 1:4 and 0:5) against *P. falciparum* 3D7. The x and y axes show the FIC_50_ values of the two mAbs taking part of the combination. The grey dashed line indicates the expected results following the definition of Loewe additivity. The purple line links the observed values of each ratio of the mAb combination that induces 50% GIA. The points below the additivity line indicates synergy, while points above indicate antagonism. The following mAb combinations were investigated: CyP1.9:CyP2.38, CyP1.9:CyP2.39 and CyP2.38:CyP2.39.

For the CyP1.9:CyP2.38 and CyP2.38:CyP2.39 mAb combinations, the isobolograms showed that the concentration of mAbs needed to neutralize 50% of parasites, was lower than what would be predicted by Loewe additivity (**Figure 4**). Both mAb combinations deviates strongly below the line of additivity, indicating a synergistic effect, also demonstrated by the combination indexes **(** **Figure S3** **)**.

In contrast, the isobologram for the CyP1.9:CyP2.39 mAb combination showed that the concentration of mAbs required to neutralize 50% of parasites, is higher than predicted by Loewe additivity **(** **Figure 4** **)**. The result indicates that the CyP1.9:CyP2.39 combination appears to be antagonistic. This is probably because these mAbs compete for the same binding site on PfCyRPA and thus the presence of CyP2.39 (the less potent mAb) dilutes the effect of CyP1.9. Despite the appearance of Bliss subadditivity in earlier experiments **(** **Figure 2A** **)**, the CyP1.9:CyP2.39 combination appears to be antagonizing by the Loewe additivity definition. The only exception is the 4:1 fixed-ratio of CyP1.9:CyP2.39, where the observed GIA falls closer to the line of additivity, indicating that the contribution of CyP2.39 is minimal at this antibody ratio. Nonetheless, the combinations of mAbs targeting different epitopes on PfCyRPA were clearly synergistic regardless of which method was used to define synergy.

### Strain-Transcending Neutralization Capacity of Anti-PfCyRPA mAb Combinations

**Figure 5:**
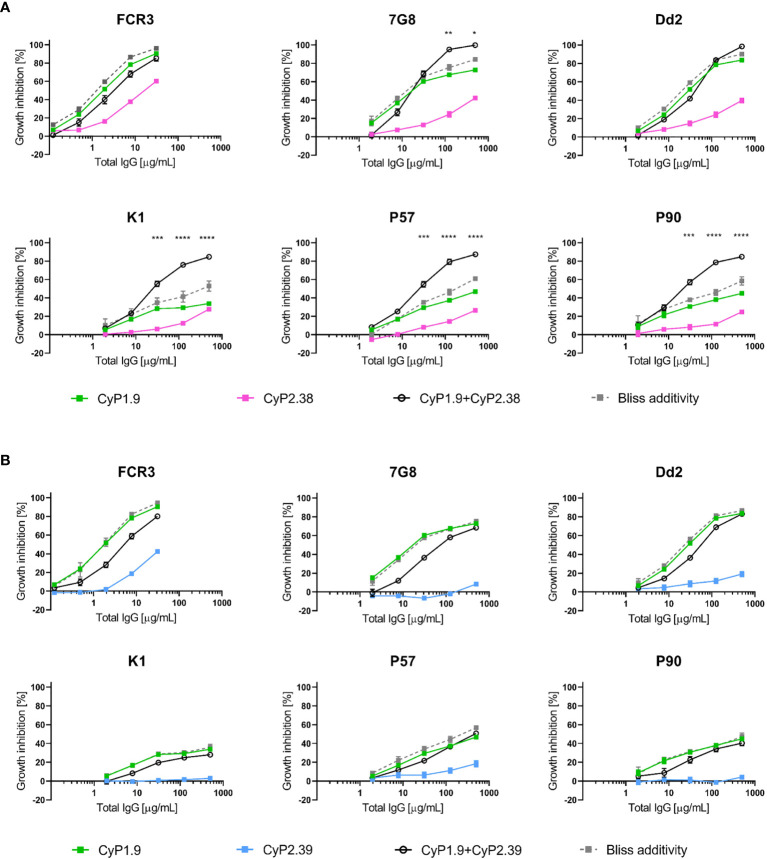
Growth inhibition activity on heterologous P. falciparum strains using anti-PfCyRPA mAb combinations. GIA assays on laboratory-adapted and clinical isolates of *P. falciparum* using mAbs **(A)** from different epitope-binning groups where CyP1.9 and CyP2.38 are mixed in a 1:1 ratio and **(B)** from the same epitope-binning groups where CyP1.9 and CyP2.39 are mixed in a 1:1 ratio. The solid black line represent the experimental values and the grey dashed line representing the predicted additive effect when combing the mAbs according to Bliss Additivity. Data points represent the mean of triplicates from two independent experiments. Error bars indicate SEM for all six replicates over two experiments. Asterisks indicate that the experimental and predicted values significantly show synergy by using a 2-way ANOVA with Bonferroni’s multiple comparison test (*p,0.05, **p,0.01, ***p,0.001, ****p,0.0001).

When the individual anti-PfCyRPA mAbs were assessed against multiple laboratory-adapted and clinical isolates of *P. falciparum*, we observed strain-dependent neutralization potencies. Therefore, we found it highly relevant to conduct a similar assessment on the combinations of anti-PfCyRPA mAbs. To detect synergy, the GIA effect of anti-PfCyRPA mAbs was measured both individually and in combination. For each combination of mAbs a predicted additive GIA effect was calculated by Bliss additivity. The GIA assays were performed utilizing the synergistic combination of CyP1.9 and CyP2.38 **(** **Figure 5A** **)** and the sub-additive combination of CyP1.9 and CyP2.39 **(** **Figure 5B** **)**.

As expected, synergy occurred when combining CyP1.9 and CyP2.38 on the majority of *P. falciparum* strains. However, when tested on FCR3 and Dd2, the mAb combination was only sub-additive or even antagonistic at some concentrations. Thus, there seems to be a strain-dependent variation of the mAb combination, which could be linked to the increased potency of CyP1.9 particularly on these strains **(** **Figure 1C** **)**. When combining mAbs that target the same area on PfCyRPA, no synergistic effect was found on any of the parasite strains, which matched the results we obtained against 3D7 **(** **Figure 2A** **)**.

### The Neutralizing Capacity of Anti-PfCyRPA mAbs Is Potentiated by Anti-PfRh5 mAbs

**Figure 6:**
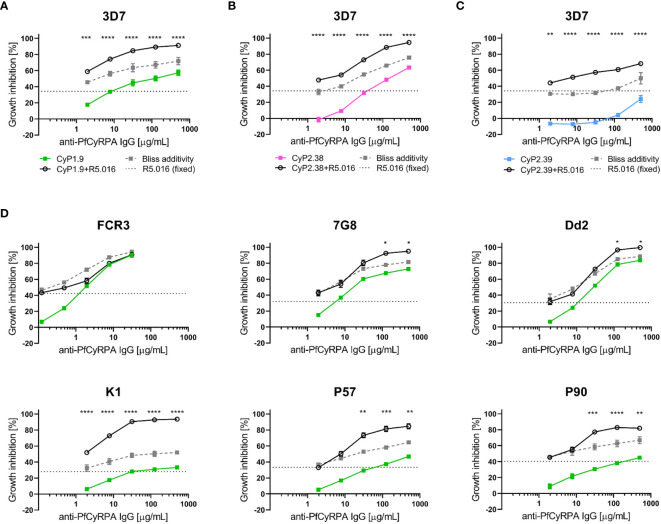
Growth inhibition activity on heterologous *P. falciparum* strains using anti-PfCyRPA and anti-PfRh5 mAb combinations. **(A)** Growth inhibitory activity against 3D7 reference strain using the anti-PfCyRPA mAb, CyP1.9, and the anti-PfRh5 mAb, R5.016. A dilution series of CyP1.9 is mixed with a fixed concentration of R5.016, which when used alone gives approximately 30-40% GIA (dotted black line). The predicted additive effects were calculated according to Bliss additivity, and are shown in the grey dashed line. The experimental data on the CyP1.9 and R5.016 mAb combination is shown in the solid black line. Similar GIA assay were performed against 3D7 using **(B)** CyP2.38 and a fixed concentration of R5.016 and **(C)** using CyP2.39 and a fixed concentration of R5.016. **(D)** GIA assays on laboratory-adapted and clinical isolates of *P. falciparum* using a dilution series of CyP1.9 and fixed concentration of R5.016. For all panels data points represent the mean of triplicates from two independent experiments. Error bars indicate SEM for all six replicates over two experiments. Asterisks indicate that the experimental and predicted values significantly show synergy by using a 2-way ANOVA with Bonferroni’s multiple comparison test (*p,0.05, **p,0.01, ***p,0.001, ****p,0.0001).

Previously, it has been shown that combinations of antibodies specific for PfCyRPA and PfRh5 have additive or synergistic effects (15, 19, 21, 33). Therefore, anti-PfCyRPA mAbs with neutralizing capacity were tested in combination with an anti-PfRh5 mAb to determine if similar improvements in neutralization occurred. The definition of Bliss additivity was used to assess the additive GIA effect of the combinations. Due to the high potency of the anti-PfRh5 mAb (R5.016), a fixed concentration was used with and without the addition of anti-PfCyRPA mAbs at a range of concentrations (26). Remarkably, mAb R5.016 potentiated the neutralizing effect of all three anti-PfCyRPA mAbs against the 3D7 parasites, showing a clear synergistic effect for each of these combinations **(** **Figures 6A–C** **)**.

Having clearly observed synergy, the functional assessment was extended to other laboratory-adapted and clinical isolates of *P. falciparum*. The CyP1.9 and R5.016 combination was capable of potentiating the neutralizing effect on the majority of *P. falciparum* strains **(** **Figure 6D** **)**. However, when tested against 7G8 and Dd2, the synergistic effects only occurred at the highest concentrations. Furthermore, when tested against FCR3, only sub-additive effects were observed. A strain-dependent tendency seems to recur, as FCR3 was less affected by the mAb combination. We speculate that this is linked to the high neutralizing capacity of the individual anti-PfCyRPA mAbs on this strain. A similar strain-dependent tendency in neutralization was observed when combining CyP2.38 or CyP2.39 with a fixed concentration of the anti-PfRh5 mAb **(** **Figure S4** **)**. These data show that the effect of mAb combinations against one strain of *P. falciparum* is not necessarily transcending to other strains, highlighting the complexity of neutralizing this parasite.

### Strain-Dependent GIA Variation Is Independent of Antigen Polymorphism and Expression Level

**Figure 7:**
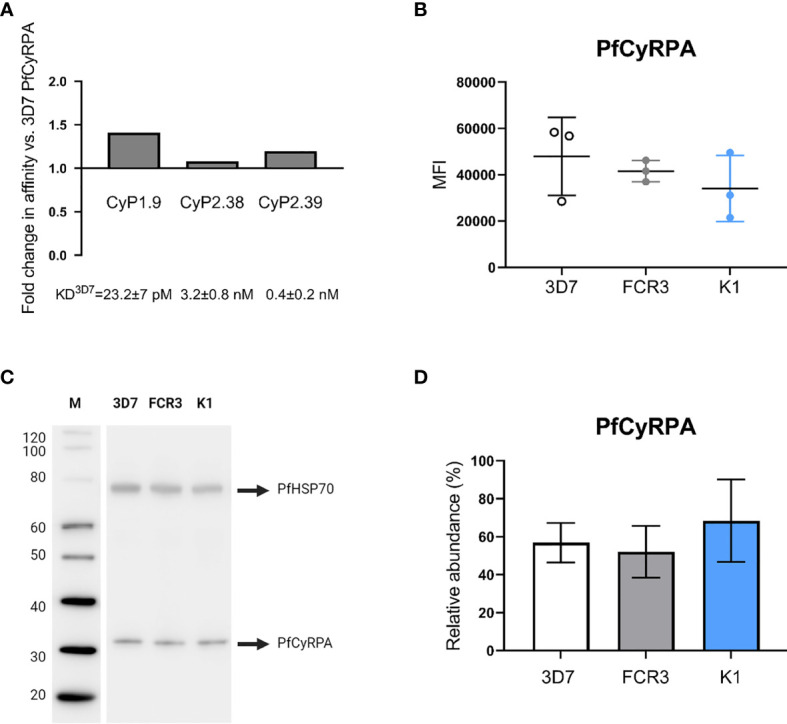
Binding to PfCyRPA (R339S) variant and endogenous expression level of PfCyRPA in three P. falciparum strains. **(A)** Real-time analysis by SPR of anti-PfCyRPA mAbs binding to PfCyRPA variant with the most common naturally occurring amino acid substitution R339S. The error bars show fold-change in affinity compared to 3D7 reference sequence PfCyRPA. **(B)** Intracellular detection of PfCyRPA analyzed by flow cytometry on fixed and permeabilized merozoites (3D7, FCR3 and K1). The merozoites were stained with the primary mAb (CyP1.9) and the secondary mAb (horse anti-mouse IgG conjugated to FITC) as well as the nuclei stain DAPI. In the scatter plot, each dot represents the geometric MFI in the FITC channel from an independent experiment, repeated on each strain three times. The mean and the SD are shown in the scatter plot. A one-way ANOVA with Tukey’s multiple comparison test was applied to determine if there was any significant difference in the expression level of PfCyRPA between the three strains. **(C)** Western blot of 3D7, FCR3 and K1 merozoite lysates (10 µg lysate per lane) showing staining of PfCyRPA using CyP1.9 and PfHSP70 (loading ctrl) using a rabbit anti-*P. falciparum* HSP70 polyclonal Ab. **(D)** Quantitative analysis of PfCyRPA amounts from two independent blots, each strain was added in triplicate on each blot. Error bars represent SD from two independent quantitated immunoblots using different batches of merozoite lysates. A one-way ANOVA with Tukey’s multiple comparison test was applied to determine if there were any significant difference in the expression level of PfCyRPA between the three strains.

To address the strain-dependent variation observed in the GIA assays, we explored whether the neutralizing mAbs recognize the most common PfCyRPA polymorphism, R339S (14, 34). The effect of the amino acid substitution was determined by measuring the affinity of each of the neutralizing mAbs to the PfCyRPA variant and comparing it with the affinity to 3D7 PfCyRPA. The results showed that none of the mAbs had a significant differential affinity to the PfCyRPA variant **(** **Figure 7A** **)**. This indicates that there is no connection between the PfCyRPA polymorphism and the strain-dependent GIA variation.

Another important factor is the amount of endogenous PfCyRPA expressed by the *P. falciparum* merozoites, as accessibility of the antibody target may correlate with the ease of neutralization. We therefore quantified the endogenously expressed PfCyRPA in 3D7, FCR3 and K1 merozoites, as the two latter strains varied the most in their susceptibility to being neutralized by the anti-PfCyRPA mAbs **(** **Figures 1C, E** **)**. PfCyRPA resides in the microneme of merozoites and is released upon invasion of erythrocytes (11, 18). To quantify the endogenous PfCyRPA expression levels, intracellular staining was performed on fixed and permeabilized merozoites analyzed by flow cytometry. Similar MFI’s were observed across the three *P. falciparum* strains **(** **Figure 7B** **)**, indicating that PfCyRPA expression levels do not significantly vary between the parasite strains.

To confirm the findings using a different approach, we performed quantitative western blotting. The amount of PfCyRPA was detected by an anti-PfCyRPA mAb (CyP1.9) on 3D7, FCR3 and K1 merozoite lysates. At the same time, PfHSP70 served as a loading control to normalize data and was detected using a rabbit anti-*P. falciparum* HSP70 Ab (StressMarq) **(** **Figure 7C** **)**. To assess the differences of PfCyRPA quantitatively, the strain-specific merozoite lysates were added in triplicates in two independent western blots, loading equimolar amounts in each lane. The PfCyRPA band intensities were measured in relative abundance to the PfHSP70 loading control. No significant differences in PfCyRPA expression were detected across the three *P. falciparum* strains **(** **Figure 7D** **)**, confirming the results obtained from the flow cytometry analysis.

## Discussion

Antibodies are key components in adaptive humoral immunity against blood-stage malaria. They function *via* multiple mechanisms, one being direct inhibition (neutralization) by which antibodies block key parasite proteins from taking part in the invasion process of erythrocytes. The discovery of PfCyRPA as a conserved antigen taking part in a non-redundant invasion pathway has highlighted the protein as a promising blood-stage vaccine candidate (11, 15, 18).

Here, we show that numerous laboratory-adapted parasite lines as well as naturally-circulating parasite isolates from Ghana are susceptible to anti-PfCyRPA mAb neutralization. The neutralizing potency against the 3D7 strain requires lower or in some cases comparable concentrations than already published anti-PfCyRPA mAbs (12, 18, 20, 32). Furthermore, we expanded upon these findings, by identifying several synergistic antibody combinations using a comprehensive quantitative methodology, which has the potential to advance pre-clinical vaccine candidate screenings.

PfCyRPA is highly conserved, with only one single nucleotide polymorphism (R339S) above 5% prevalence (14). The laboratory-adapted parasite lines FCR3 and K1 were included in the functional assessment of our mAbs as both carry this polymorphism. The clinical *P. falciparum* isolates from Ghanaian patients were sequenced, however none carried this common amino acid substitution. The functional assessment showed that only three of the anti-PfCyRPA mAbs were neutralizing on their own. All parasite strains were susceptible to anti-PfCyRPA mAb neutralization, however, the potency seemed to be strain-dependent, maintaining a similar hierarchy of the mAbs. Remarkably, FCR3 and K1 vary the most in their susceptibility to be neutralized by anti-PfCyRPA mAbs. A possible link between PfCyRPA polymorphism and affinity was investigated, however no apparent relationship was observed, indicating that the polymorphism is unconnected to the potency of anti-PfCyRPA mAbs in the GIA assay. It is likely that the fundamental need to interact with PfRh5 and PfRipr imposes constraints on the extent of mutations in PfCyRPA, which might reduce the risk of evolving resistance by antigenic drift.

Further characterization of the anti-PfCyRPA mAbs grouped them into green or pink epitope bins. Surprisingly both bins contained neutralizing and non-neutralizing anti-PfCyRPA mAbs, demonstrating that targeting a domain comprising protective epitopes does not necessarily result in a neutralizing capacity. All three mAbs in the green bin recognized blades 2 and 3 (fragment A) on PfCyRPA, which includes a previously well-characterized protective epitope (19, 20), however, only two of these mAbs were neutralizing. The kinetic analysis of the anti-PfCyRPA mAbs, suggests that antibody association-rate, as opposed to affinity, is a key indicator of antibody potency. A possible explanation for this relationship may be that PfCyRPA is an intracellular antigen only accessible to direct antibody inhibition during the short period of invasion, resulting in a limited time window for the mAbs to associate with their target (35). The importance of high antibody association-rates has also been found against other merozoite antigens (26, 36), emphasizing the importance of addressing this parameter for generating efficacious blood-stage malaria vaccines. The identification of protective epitopes in blades 2 and 3, suggests that this fragment may induce a higher ratio of neutralizing antibodies compared to full-length PfCyRPA when used as an immunogen. Nevertheless, it is encouraging that multiple independent studies have identified blades 2 and 3 as harboring protective epitopes (19, 20).

The anti-PfCyRPA mAbs in the pink epitope bin were shown to target overlapping epitopes on PfCyRPA, involving blades 4 and 5. The protective epitope of the neutralizing mAb, CyP2.38, was thought to be slightly different, as it was highly dependent on interactions with other blades on PfCyRPA, as no reactivity was observed with fragment E. Thus, CyP2.38 revealed a novel protective epitope on PfCyRPA. Furthermore, as the association-rate of CyP2.38 was 8-10 fold higher compared to other mAbs in this bin, we cannot diminish the importance of this parameter. Hence, we found that the neutralizing capacity of the anti-PfCyRPA mAbs is determined by both epitope and association-rate.

Earlier studies have shown that antibody potency in the *in vitro* GIA assays correlate with results using *in vivo* models (18, 37–39). However, high antibody concentrations are required for *in vivo* protection, which might represent a hurdle for the development of a PfCyRPA-based vaccine. Here, we show that synergy occurs between pairs of mAbs targeting different epitopes on PfCyRPA. In addition, antibody CyP2.27 which had negligible neutralization on its own, still synergized in combination with non-competing neutralizing antibodies. These results encourage the rational design of a PfCyRPA vaccine that comprises multiple protective epitopes, as lower titers of total PfCyRPA specific antibodies may be required to confer protection.

By utilizing Bliss additivity and Loewe additivity definitions, non-competing neutralizing mAbs were shown to strongly synergize in inhibiting the growth of 3D7 parasites. The synergistic effect applied to multiple laboratory-adapted and clinical isolates of *P. falciparum*. However, this cooperation between mAbs was not transcending to all parasites lines. Throughout this study, no mAb combinations were able to synergize against FCR3 parasites. Even when combining the anti-PfCyRPA mAbs with a very potent anti-PfRh5 mAb, no synergy was observed. It is likely that this tendency is linked to the high neutralization capacity of the individual anti-PfCyRPA mAbs on this parasite strain. The quantitative assessment suggests that although PfCyRPA is highly conserved and takes part in a non-redundant invasion pathway, it does not necessarily facilitate similar neutralization activities in *in vitro* GIA assays.

To investigate the strain-dependent GIA assay variation, the endogenous expression level of PfCyRPA was evaluated on three *P. falciparum* strains, as the accessibility of the antibody target might correlate with the ease of neutralization. Previous studies have shown that differential expression levels of merozoite antigens provide a mechanism for phenotypic variation in erythrocytes invasion by *P. falciparum* parasites (40–42). Therefore we evaluated if similar applied to PfCyRPA. However, neither the flow cytometry analysis nor quantitative western blotting detected significant differences in PfCyRPA expression levels in merozoites. This suggests that this parameter is unlinked to antibody potency and that other parameters are expected to be essential for evaluating blood-stage malaria vaccine candidates.

The kinetics of merozoite invasion could explain the *in vitro* differences. An earlier study has shown that the number of erythrocytes contacted before invasion vary significantly between *P. falciparum* strains (10). Another study shows that the egress of merozoites before initiating invasion can take all from 2-90 seconds (43). These differences could explain the strain-dependent variation observed here, especially as the mechanism of direct antibody inhibition is so dependent on antibody association-rates (26). It is generally assumed that the duration of the three steps of merozoite invasion (pre-invasion, invasion and echinocytosis) are conserved across *P. falciparum* strains. However, the number of studies on this topic are few and have only been performed on a limited number of parasite lines (10, 43).

In summary, this study provides further evidence that PfCyRPA is an attractive candidate for developing a second-generation malaria vaccine. Anti-PfCyRPA mAbs effectively neutralized laboratory-adapted parasite lines as well as short-term adapted parasite isolates from Ghana. We provided a clear demonstration of synergy when targeting multiple protective epitopes on PfCyRPA simultaneously. Furthermore, combinations of anti-PfCyRPA and anti-PfRh5 mAbs were shown to give rise to synergistic interactions. However, strain-dependent variability in neutralizing potency was observed, which was seemingly independent of PfCyRPA polymorphism and expression level. It remains unknown whether these *in vitro* GIA differences are relevant to *in vivo* neutralization. Nevertheless, the lack of clear synergistic effect when combining antibodies in the GIA assays, particular against FCR3 parasites, demonstrates the difficulties in selecting components for multi-antigen vaccines. To utilize the full potential of synergizing antibodies to confer protection against malaria, our data suggest that antigens will not only need to be conserved at the sequence level, but also in their functional importance to the parasite. Finally, this work identified protective regions on PfCyRPA, paving the way to test these fragments alone or in combination with PfRh5 (or other merozoite antigens) in order to improve the efficacy of blood-stage malaria vaccines.

## Data Availability Statement

The original contributions presented in the study are included in the article/**Supplementary Material**. Further inquiries can be directed to the corresponding author.

## Ethics Statement

The animal study was reviewed and approved by Danish Animal Experiment Inspectorate.

## Author Contributions

ASK and LB designed experiments. ASK, KB, MB, AK, and BC conducted experiments. ASK, MW, and LB performed data analysis. LB, AJ, and MB supervised experiments. ASK and LB wrote the first draft of the manuscript. All authors contributed to the article and approved the submitted version.

## Funding

The work was supported the Novo Nordisk Foundation (NNF170C0026778) as well as Danida (17-02-KU and 12-081RH).

## Conflict of Interest

The authors declare that the research was conducted in the absence of any commercial or financial relationships that could be construed as a potential conflict of interest.

## Publisher’s Note

All claims expressed in this article are solely those of the authors and do not necessarily represent those of their affiliated organizations, or those of the publisher, the editors and the reviewers. Any product that may be evaluated in this article, or claim that may be made by its manufacturer, is not guaranteed or endorsed by the publisher.
