# Supplementary figures and images for: Strain-Dependent Inhibition of Erythrocyte Invasion by Monoclonal Antibodies Against Plasmodium falciparum CyRPA
Source: Front Immunol. 2021 Aug 10;12:716305. doi: 10.3389/fimmu.2021.716305 (PMC8383283; doi:10.3389/fimmu.2021.716305)

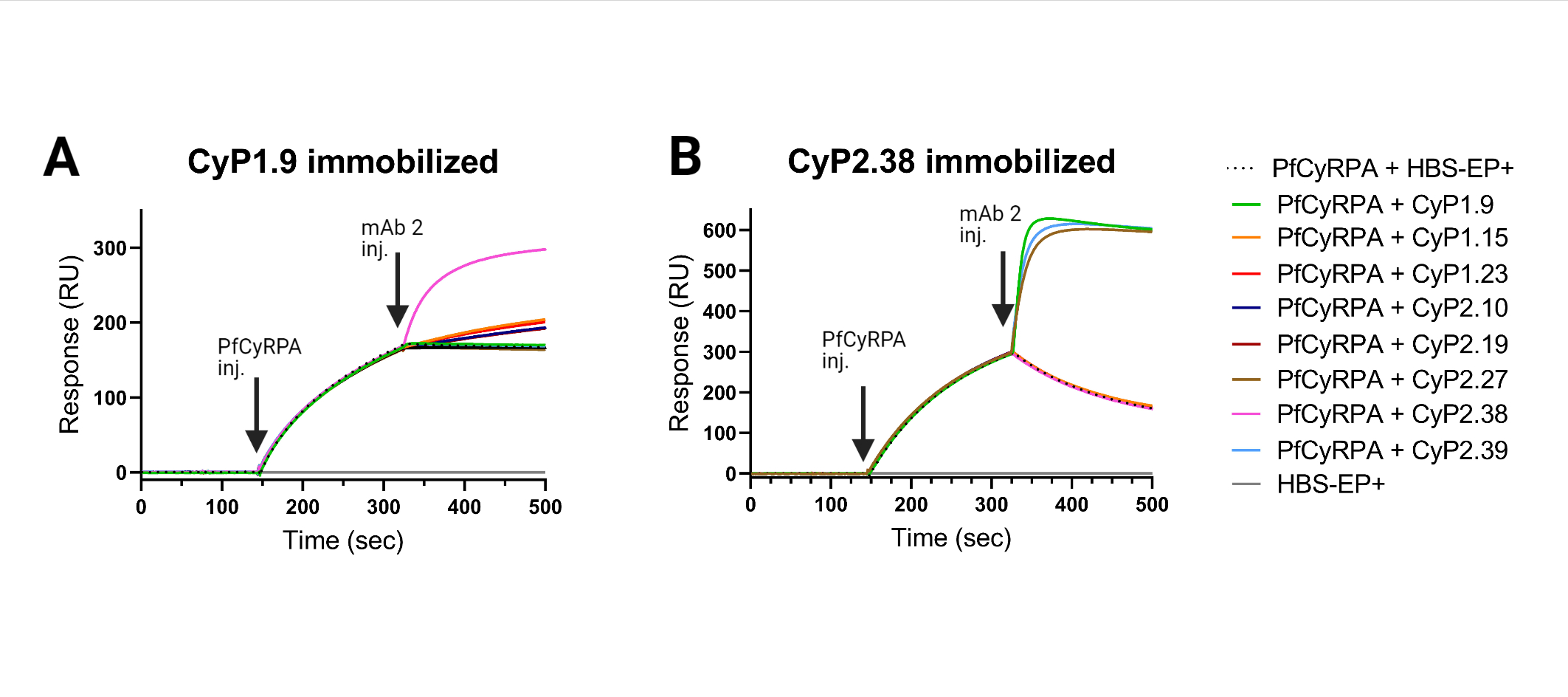

Supplement: Supplementary file 1 [file Image_1.jpeg]

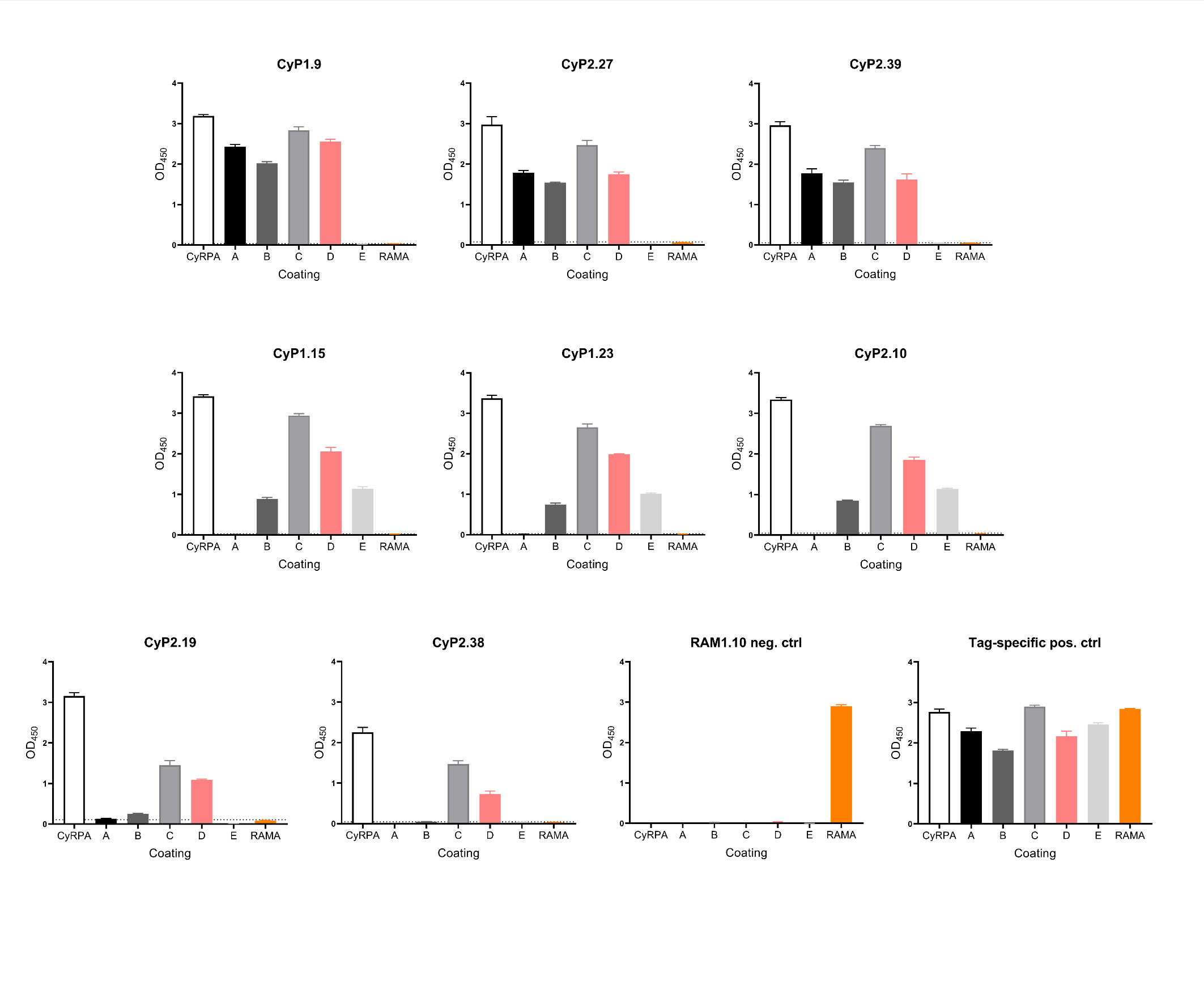

Supplement: Supplementary file 2 [file Image_2.jpeg]

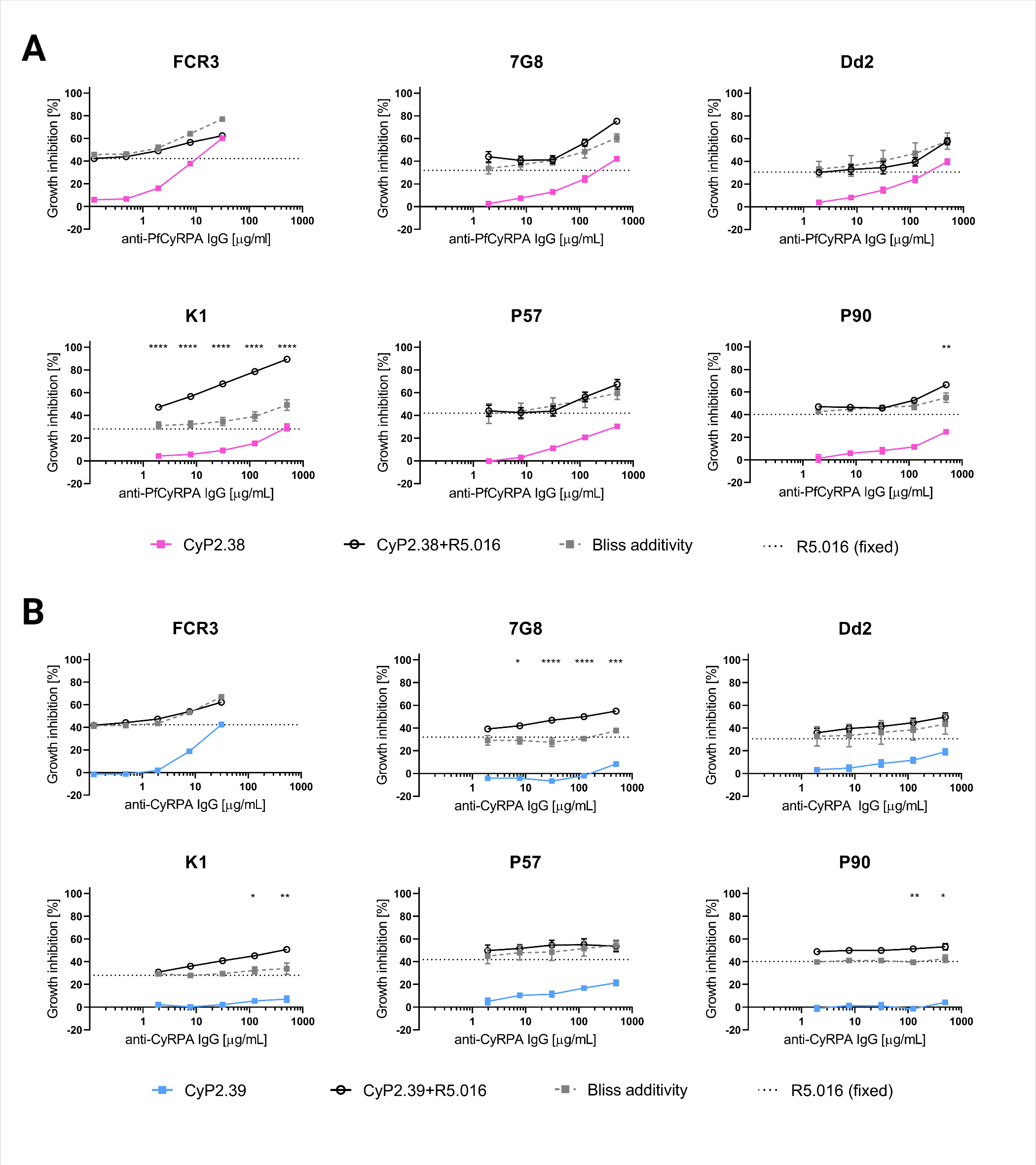

Supplement: Supplementary file 4 [file Image_4.jpeg]
