# Supplementary material for: Strain-Dependent Inhibition of Erythrocyte Invasion by Monoclonal Antibodies Against Plasmodium falciparum CyRPA
Source: Front Immunol. 2021 Aug 10;12:716305. doi: 10.3389/fimmu.2021.716305 (PMC8383283; doi:10.3389/fimmu.2021.716305)
Supplement: Supplementary file 3 [file Image_3.pdf]

mAb combination and ratio	Combination Index
CyP1.9 & CyP2.38 (4:1)	0.060
CyP1.9 & CyP2.38 (3:2)	0.055
CyP1.9 & CyP2.38 (2:3)	0.056
CyP1.9 & CyP2.38 (1:4)	0.072
CyP1.9 & CyP2.39 (4:1)	0.79
CyP1.9 & CyP2.39 (3:2)	2.17
CyP1.9 & CyP2.39 (2:3)	3.54
CyP1.9 & CyP2.39 (1:4)	2.80
CyP2.38 & CyP2.39 (4:1)	0.104
CyP2.38 & CyP2.39 (3:2)	0.087
CyP2.38 & CyP2.39 (2:3)	0.087
CyP2.38 & CyP2.39 (1:4)	0.104
