# Supplementary material for: Strain-Dependent Inhibition of Erythrocyte Invasion by Monoclonal Antibodies Against Plasmodium falciparum CyRPA
Source: Front Immunol. 2021 Aug 10;12:716305. doi: 10.3389/fimmu.2021.716305 (PMC8383283; doi:10.3389/fimmu.2021.716305)
Supplement: Supplementary file 5 [file DataSheet_1.pdf]

Supplementary information – Figure legends

Figure S1. Epitope-binning by classical sandwich blocking using SPR systems.
The two neutralizing mAbs **(A)** CyP1.9 and **(B)** CyP2.38, were immobilized in different flow cells on the same CM5 chip. The first injection is the PfCyRPA antigen, the second injection is the competing mAb, both injections are indicated by an arrow. An increment in RU at the second injection site is indicative of a none-competing mAb.

Figure S2. Reactivity to PfCyRPA fragments using the panel of PfCyRPA-specific mAbs
Reactivity of the anti-PfCyRPA mAbs (5 µg/mL) to different fragments of PfCyRPA (2 µg/mL) by ELISA. A mouse anti-PfRAMA mAb (RAM1.10) served as a negative control, while a tag-specific mouse mAb served as a positive control. Positive cut-off are represented by a black dashed line, calculated as the mean + 3SD of OD values in binding to irrelevant antigen (RAMA).

Figure S3: Combination indexes for anti-PfCyRPA mAb combinations

Dose-response data were analysed to interpolate EC_{50} values, which then were used to calculate two FIC_{50} values for each mAb ratio tested. The sum of the two FIC50 values is defined as the combination index (CI). CI < 1, CI=1, and CI > 1 respectively indicate synergy, additivity and antagonism.

Figure S4. Growth inhibition activity on heterologous *P. falciparum* strains using anti-PfCyRPA and anti-PfRh5 mAb combinations.
(A) GIA assays on laboratory-adapted and clinical isolates of *P. falciparum* using the CyP2.38 and R5.16 mAb combination. A dilution series of CyP2.38 is mixed with a fixed concentration of R5.16, when used alone gives approximately 30%-40% GIA (dotted black line). The predicted additive effects were calculated according to Bliss Additivity, and are shown in the grey dashed line. The experimental data on the CyP2.38 and R5.16 mAb combination is shown in the solid black line. **(B)** Similar GIA assays utilising the CyP2.39 and R5.16 mAb combination. Data points represent the mean of triplicates from two independent experiments. Error bars indicate SEM for all six replicates over two experiments. Asterisks indicate that the experimental and predicted values significantly show synergy by using a 2-way ANOVA with Bonferroni's multiple comparison test (*p,0.05, **p,0.01, ***p,0.001, ****p,0.0001).
